# Beyond non-technical skills: training ophthalmologists to appraise and integrate emerging technologies

**DOI:** 10.1038/s41433-025-04017-4

**Published:** 2025-09-20

**Authors:** Thomas A. Whitelaw, Evie Fioratou

**Affiliations:** School of Medicine, University of Dundee, Dundee, UK

**Keywords:** Education, Technology

## Abstract

As technology becomes increasingly complex and autonomous, ophthalmologists need to know the capabilities and limits of their tools.

Ophthalmology is technologically intense, with tools ranging in complexity from the direct ophthalmoscope to optical coherence tomography (OCT), AI imaging and advanced surgical systems. As innovation accelerates the ophthalmologist’s role is expanding. As well as clinical expertise, understanding use-cases, facilities and limitations of many technologies are now required. AI systems, with their hallucinations and “black box” thinking, highlight the importance of this. Their contextual limits, validation and the need to override them with clinical judgement, require knowledge and expertise [1].

A “full understanding” of ophthalmic tools would entail expertise in materials science, semiconductors, atomic structure and fundamental physics. An unattainable objective. Yet, how tools behave, their optimal integration into workflows, adaptability and usability can all be mastered. These describe Human Factors (HF), the science of understanding the relationships between humans, their working environment, and the systems, tools, and processes involved.

There is, however, a common erroneous tendency to only equate HF with non-technical skills (NTS), which are well taught within ophthalmology. The “Human Factors in intra-operative Ophthalmic Emergencies Scoring System” (HUFOES) [2] assesses trainees on domains including professionalism, teamwork and communication, while they manage simulated surgical complications. The HUFOES score renders these NTS measurable, enabling evaluation and improvement.

The London Pilot Immersive Simulation Programme is analogous [3]. This feasibility study investigated evaluation of NTS through simulation. Realistic theatre scenarios based on real cases were employed and established HF frameworks like the “Anaesthetists’ Non-Technical Skills” (ANTS) and “Non-Technical Skills for Surgeons” (NOTSS) were used to score trainees. Task management, teamwork, decision making and situational awareness measures, gave reliable objective results.

There is now an opportunity for the Ophthalmology Specialty Training (OST) curriculum to include *technical* HF teaching, such as appraisal and assessment of ophthalmic devices and technologies. Evidence shows that ophthalmology is adopting this approach already. Researchers at Moorfields Eye Hospital applied a user-centred approach to design a home administered OCT system. 81% of patients found the prototype easier than smartphones to use, while 86% found this approach appealing. Even first-time users with visual impairment completed full testing [4]. User feedback early during development enhanced design.

HF principles have shaped the design of pre-filled syringes (PFS) for intravitreal faricimab [5]. Older vials involved multiple steps, each being a potential source of error. The new PFS reduces cognitive load and simplifies workflow. Simulated use accorded 86.7%-100% pass rates with minimal error. For 35 clinical injections, no inoculation errors occurred, despite no prior user training.

These successes occurred outside the formal OST curriculum. Inclusion of technology appraisal teaching would set the foundation for future developments, preparing ophthalmologists to work safely and completely in the increasingly complex socio-technical workplace.

## Data Availability

Data sharing not applicable to this article as no datasets were generated or analysed during the current study.
